# Data of physical and electrochemical characteristics of calendered NMC622 electrodes and lithium-ion cells at pilot-plant battery manufacturing

**DOI:** 10.1016/j.dib.2023.109798

**Published:** 2023-11-10

**Authors:** Mona Faraji-Niri, Marc Fransic V. Hidalgo, Geanina Apachitei, Daniela Dogaru, Michael Lain, Mark Copley, James Marco

**Affiliations:** a Warwick Manufacturing Group, University of Warwick, CV4 7AL Coventry, UK; b The Faraday Institution, Quad One, Harwell Science and Innovation Campus, Didcot, UK

**Keywords:** Lithium-ion battery, Electrode manufacturing, Pilot-plant, Calendering process, Electrochemical characteristics, SEM/EDS images, Battery cycling, Machine learning

## Abstract

The data reported here was prepared to study the effects of calendering process on NMC622 cathodes using a 3-3-2 full factorial design of experiments. The data set consists of 18 unique combinations of calender roll temperature (85 °C, 120 °C, or 145 °C), electrode porosity (30%, 35%, or 40%), and electrode mass loading (120 g/m² or 180 g/m²). The reported physical characteristics of the electrodes include thickness, coating weight, maximum tensile strength, and density. The electrochemical performances of the electrodes were obtained by testing coin cells. In this context, 54 half-cells were produced, 3 per each calendering experiment to ensure repeatability and reliability of the results. The responses of interest included, charge energy capacity at C/2, C/5, discharge energy capacity at C/20, C/5, C/2, C, 2C, 5C, 10C, gravimetric capacity (charge at C/2, C/5, discharge at C/20, C/5, C/2, C, 2C, 5C, 10C), volumetric capacity (charge at C/2, C/5, discharge at C/20, C/5, C/2, C, 2C, 5C, 10C), rate performance (5C:0.2C), area specific impedance (at 10% to 90% state of charge (SoC) in 10 breakpoints), long-term cycling capacity (charge at C/5 for 50 cycles, discharge at C/2 for 50 cycles), long-term cycling degradation (at C/2 during 50 cycles of charge and discharge), and cycling columbic efficiency (50 cycles of C/2 charge and discharge). The details of the experimental design that has led to this data as well as comprehensive statistical analysis, and machine learning-based models can be found in the recently published manuscripts by Hidalgo et al. and Faraji-Niri et al. [1,2].

Specifications Table

**Table:** 

Subject	Electrical and Electronic Engineering
Specific subject area	Electrode manufacturing and characterization of lithium-ion batteries
Data Format	Raw Analyzed
Type of data	- Table (.xlsx) - Image (.png)
Data Collection	A set of 18 experiments were prepared using design of experiments. The specific design was a 3-3-2 full factorial, where roll temperature and porosity were at 3 levels and mass loading were set to 2 levels.Electrode and cell manufacturing data - The active material for cathode has been NMC622. - Anode is lithium metal in half-cell. - Coating and drying of the electrodes were performed in a Megtech Systems pilot-plant convective coater. - The mass loadings were recorded using MeSys scanning systems. - The porosities of the (1) pre-calendered electrodes, (2) post-calendered electrodes, and (3) discs for electrochemical testing were calculated by the Eq. (1). - The densities of the (1) pre-calendered electrodes, (2) post-calendered electrodes, and (3) discs were calculated by the Eq. (2). - The coating thicknesses of the (1) pre-calendered electrodes, (2) post-calendered electrodes, and (3) discs for electrochemical testing were measured using digital thickness gauge (Mitutoyo) at various locations. - Temperature is the reading of the calendering machine (IMC, Innovative Machine Corp.). Electrochemical testing and determinations The electrochemical data was obtained in a temperature-controlled chamber at 25 °C. The testing protocol involved upper and lower cut-off voltages of 4.2 V and 2.5 V, respectively. Formation cycle was performed at C/20 rate (charge and discharge), followed by five conditioning cycles at C/5 (charge and discharge). Discharge C-rate capacities were measured with all charging cycles performed in between at C/5 rate. The area specific impedance was measured via pulse tests. - (1) Charge and discharge energy capacities of 54 half-coin cells at different rates, (2) cycling capacity of cells over 50 cycles and (3) using a Biologic BCS-805 cycler. - Gravimetric capacities, volumetric capacities, rate performances and first cycle loss were calculated from the capacity data given the active material weight, as described by Eqs. (3) and (4). - Area specific impedances (ASI) was calculated by Eq. (5) at various SoCs through a pulse test.
Data source location	Institution: Warwick Manufacturing Group (WMG), Energy Innovation Centre. University of Warwick City: Coventry Country: United Kingdom GPS coordinates for collected samples/data: 52.38363378953185, −1.5615186436655097.
Data accessibility	Repository name: Mendeley Data Data identification number: 10.17632/wwhm2frfmy.1 Direct URL to data: https://doi.org/10.17632/wwhm2frfmy.1
Related research article	Hidalgo, M.F., Apachitei, G., Dogaru, D., Faraji Niri, M., Lain, M., Copley, M. and Marco, J., (2023) Design of Experiments for Optimizing the Calendering Process in Li-Ion Battery Manufacturing. Journal of Power Sources 573: 233091. https://doi.org/10.1016/j.jpowsour.2023.233091

## 1 Value of the Data

• This data set contains (1) the operating conditions & parameters used to coat and calender the NMC622 electrodes and (2) the relevant physical properties of the electrodes (before and after calendering) as well as the electrochemical performance of these electrodes when used in Li-ion half coin cells.
• Calendering is a key step in the Li-ion battery manufacturing process, it is the last step of manufacturing process that is still controllable. This data set tracks how changes in calendering conditions correlate with changes in the physical properties of intermediate products of the line (electrodes) and electrochemical performance of final products (battery cells).
• These data are related to the electrodes were prepared at the pilot line; the scale of manufacturing process variables compared to a real volume scale production makes the data quite transferrable to industrial-scale applications compared to laboratory-scale data.
• The data utilizes common parameters and measurements in the li-ion battery manufacturing line, making comparisons with other studies, or combining with other data sets easier.

## 2 Data Description

### 2.1 Electrode manufacturing data

The data provided in the link to the repository for this submission includes 4 folders, “Tables” to hold tabular data of intermediate and final products of manufacturing, “Megtec and MeSys” to hold the equipment's recordings, and “Biologic” to hold the cycling data, and “Images” for SEM and EDS” data.

In the Tables Folder, the file “Intermediate measurements during calendering.xlsx” tracks changes in the key parameters as the electrodes were calendered. This file has two sheets, “Cathode and “Cathode-Intermediate”. Each sheet lists the mean values of the measurements. The standard deviations are also listed in the tables below the mean values tables, but there are only available and applicable in some of the variables and not all. While “Cathode” sheet gives the data after each experiment is finished, the “Cathode-Intermediate” sheet contains the data comparing the parameters before calendering and after each pass of the calender before things are finalized. At some experiments the electrodes were passed through the calender more than once to achieve the desired porosity.

For both sheets, the “Electrode ID” column contains the ID of each experiment, with the parameters associated with that run under the “Calendering Conditions” columns. The ID names of the cathodes are in the format “NEX_CAT_240_X_Y_Z”. Here, X represents the mass loading and is either L (low, ∼120 g/m²) or H (high, ∼180 g/m²). Y represents the calender roll temperature ( °C) and is 85, 120, or 145. Z represents the porosity and is set as P (porous, ∼40%), M (medium, ∼35%), or D (dense, ∼30%).

The listed calendering parameters are:

• Target coating weight (GSM)
• Roll Temperature (of Calender) (°C)
• Calculated target porosity (%)

In Each sheet, the Before Calendering and Calendered columns compare the responses before and after the calendering passes were completed. The reported responses are:

• Coating thickness from sheet (um)
• Coating thickness from discs (um)
• Coating weight from discs (gsm)
• Density from discs (g/cm3)
• Porosity from discs (%)
• Electrode max. tensile strength (kPa)
• Roll gap (um)- includes 2 shims of ∼500 um
• Number of passes

The sheet related end of experiment data, Cathode Sheet, include variables collected from SEM/EDS images, these are:

• Gradient in carbon (% %-1)
• Gradient in fluorine (% %-1)
• *Z*1-1 value in carbon
• *Z*1-1 value in fluorine
• Moran *I* score in carbon
• Moran *I* score in fluorine
• Observed cracks in particles (Y/N)

### 2.2 Electrochemical Performance Data

The files “Half-cell (Cathode) Electrochemical Performance.xlsx” contains the data regarding the electrochemical performance of the cells. The raw data of the performance via cyclers is shared in the Biologic Folder.

This file contains the electrode level variables for each disc that has gone into the cell at the beginning and continues with the electrochemical data towards the end. Each sheet is related to an experiment in the row of the file “Intermediate measurements during calendering.xlsx”, 18 sheets for 18 experiments. Here each sheet has various columns, and each column is one of the 3 cells made from the electrodes of that particular experiment.

Specific responses electrochemical of interest for each cell (including averages and standard deviations) are:

• Discharge capacities (mAh) at C/20, C/5, C/2, 1C, 2C, 5C and 10C
• Discharge gravimetric capacities (mAh/g) at C/20, C/5, C/2, 1C, 2C, 5C and 10C
• Discharge volumetric capacities (mAh/cm³) at C/20, C/5, C/2, 1C, 2C, 5C and 10C
• Charge capacities (mAh, mAh/g, and mAh/cm³) at C/20 (for the C/20 runs) and C/5 (for all other runs)
• Rate performance at 5C:0.2C
• First cycle loss (%)
• Area specific impedance at state of charge (SoC) of 90%, 80%, 70%, 60%, 50%, 40%, 30% 20% and 10% for both charge and discharge at 5 s pulses.
• Area specific impedance at state of charge (SoC) of 50% and 10% for both charge and discharge at 2- and 30-seconds pulses (the last two rows).
• Electrode-disc specific parameters such as thickness (µm), area (cm²), coating mass (g), density (g/cm³), porosity (%), and active mass (g)

The “Half-cell Electrochemical Performance Cycling Performance.xlsx” file contains the long-term cycling performance of each cell (50 cycles). The responses of interest are as below at given Crates. The first row is the cell ID that correlated to the previously described excel file of “Half-cell (Cathode) Electrochemical Performance.xlsx”. The second row in this file correlated back to the experiments listed in the rows of “Intermediate measurements during calendering.xlsx”.

• Discharge capacity
• Charge capacity
• Discharge capacity degradation
• Columbic efficiency

The folder “Images”, contains a file named “Calendering SEM+EDS.docx”. This file includes all the SEM images, the EDS maps and the detailed calculations from those.

The Calendering Test Matrices include the labels for each experiment and is summarizing the experimental conditions, same reported in excel file of “Intermediate measurements during calendering.xlsx” for ease of access. The Spatial Autocorrelation Measurements are given in two tables for Carbon, Fluorine and Sodium based on the EDS maps. All tables up to this point are separate for high and low coat weights for better clarity.

The images of SEM and EDS are then given in the rest of this file. Each image block refers to one of rows of the labeling tables at the first page. The images are before (Left) and after (Right) calendering and the tag at the left bottom of each image in there refer to NC (non calendered), C (Calendered), L/H for low or high coat weights, and 00XX refers to the sheet number. For example, the first SEM image at the left of the first image block, has the tag of NC240_L01_0002 and therefore is related to non calendered electrode of low coat weight table, sheet number 2 (L-Sheet 2) which is the second row of the first table.

Each image block includes the Carbon and Fluorine maps as well as the graphs from which the Carbon and Fluorine Gradient is calculated. The last two tables here are referring to the results of element distribution analysis.

## 3 Experimental Design, Materials and Methods

### 3.1 Design of experiments

The design space was prepared using a 3-3-2 full factorial in Stat -Ease Design Expert Software V 22.0.2. The calender roll temperature was 85 °C, 120 °C, or 145 °C, porosity was 30%, 35% or 40% and GSM (g/m²) was either 120 g/m² or 180 g/m².

### 3.2 Electrode manufacturing methods

Mixing of the slurry was performed using a 1L Eirich mixer. The composition was 96% active material (NMC622), 2% binder (PVDF 5130, Solvay) and 2% conductive additive (C65, Imerys). NMP was used as solvent to obtain a final solid content of 67%.

A pilot line coater (Durr Megtec) was used to coat the electrodes. The line utilized a reverse comma bar coater assembly and integrated 3-zone drying oven. The target mass loadings were ∼2 and 3 mAh/cm² and were achieved by varying the comma bar gap. The coating ratio and line speed were set to 130% and 1 m/min, respectively. The drying ovens were set to 85 °C (zone 1), 110 °C (zone 2) and 95 °C (zone 3) and the air speed was set at 7.5 m/s.

A lab calender (Innovative Machine Corporation) with roll diameter of 203 mm was used to calender the electrodes. The temperature for the calender rolls was set as 85 °C, 120 °C, or 145 °C. Stainless steel shims (pre-heated to the same temperature as the rolls) were used to reduce the difficulties of calendering thin electrodes. Electrodes were placed between the shims and immediately passed through the heated calender rolls. This was done 1-3x to the ∼28 × 11 cm electrodes until the thickness is +/-1 um within the target. Thickness was measured at 15 different points in all the stages of calendering: initial (before calendering), intermediary (all passes) and final (after calendering). The roll gap is used to control the pressure applied on the electrode. The line speed and hydraulic pressure set point were set at 0.8 m/min and 4000 psi, respectively. The porosity of each electrode is calculated as: 1 – (density of electrode / theoretical density at 0% porosity).

### 3.3 Electrochemical testing methods

After calendering, electrodes were cut into discs (1.48 cm diameter) using EL Cell cutters. The mass and the thickness of each individual disc were recorded. The electrodes were used in half cells (2032 coin cells) with a Li metal counter electrode (15 mm diameter disc), assembled in a glove box with an Argone atmosphere. The separator used was 16 mm discs of Celgard H1609 microporous tri-layer membrane (PP/PE/PP). The electrolyte formulation is 1M LiPF_6_ in EC:EMC 3:7 (vol.) + 1wt% VC. 80 µl of electrolyte was used per cell. Spacers with the thickness of 1 mm in total and wave springs were also used in the cell assembly.

The cell tests involved a sequence of charge and discharge cycles at different rates. They begin with formation (± C/20), conditioning (five cycles at ± C/5), then rate tests (charge at C/5, successive discharges at C/5, C/2, 1C, 2C, 5C, and 10C). The gravimetric capacities (mAh/g) and volumetric capacities (mAh/cm³) were calculated using the discharge capacities normalized to the weight of active material and volume of the electrode, respectively.

### 3.4 Data collection methods

Coating conditions (comma bar gap, coating ratio, web speed, drying air temperature and drying air speed) were set and recorded by the Megtech Systems coater. Mass loadings of dry electrodes were recorded by the MeSys GmbH system. The Mass loading was also measured for each disc cut from the coating sheets after the drying process. The porosities were calculated using electrode densities. Coating thicknesses were recorded manually (Mitutoyo). Electrodes maximum tensile strength was also measured manually by using an adhesion tester from Zwick Roell, Z0.5 and via 180 pull method.

Carbon and fluorine distribution were determined by spatial autocorrelation and join counting *Z*-1 1 value and Moran *I* score from the scanning electron microscope (SEM) and X-ray spectroscopy (EDS) images that were collected from a Hitachi TM3030 microscope fitted with a 30 mm 2 energy dispersive detector (Oxford Instruments). Calculations are conduced by Eqs. (6 and 7).

Electrochemical testing data was collected from .mpt files from Biologic cycler software.

## 4 Calculations

The electrode and disc porosity has been calculated via (1) with the Density given as (2). Here, *TDensity* (g/cm^3^) is the theoretical density at 0% porosity and *CAM* (mAh/g) is the capacity of active material when in powder form. The *TDensity* for all cases of this study has been 4.458 (g/cm^3^).

(1) $$Porosity\left(\%\right)=\left(1-\frac{Density(g/cm^{3})}{TDensity(g/cm^{3})}\right)*100$$

(2) $$Density\left(g/cm^{3}\right)=\frac{Coating\;Weight(g/m^{2})}{Coating\;Thickness(um)}$$

Gravimetric and volumetric capacities were calculated by the following equations of (3) and (4).

(3) $$Gravemetric\;Capacity\left(mAh/g\right)=\frac{Capacity(mAh)}{\left(Electrode\;Mass(g)-Foil\;Mass(g)\right)\left(Active\;Material(wt\%)\right)}$$

(4) $$Volumetric\;Capacity\left(mAh/cm^{3}\right)=\frac{Capacity(mAh)}{Cell\;Area\left(cm^{2}\right)\left(Total\;Thickness(um)-Foil\;Thickness(cm)\right)}$$

The ASI has been calculated by running pulse tests through (5). Here *V_t1_* and *I_t1_* are the voltage and current of the half-cells at the end of each pulse, and *V_t2_, I_t2_*, are the measured values right after the applied pulse.

(5) $$Impedance\left(\Omega \right)=\left(V_{t_{1}}\left(V\right)-V_{t_{2}}\left(V\right)\right)/\left(I_{t_{1}}\left(A\right)-I_{t_{2}}\left(A\right)\right)$$

The current pulses applied were 10  s wide. Two of the ASI measurements at 50% and 20% SoC were repeated using pulse widths of 2  s and 30  s as well.

To get the *Z*1-1 scores, the EDS maps of carbon and fluorine were first decolored to black and white to improve the contrast of pixels. The images were then converted to text files using ImageJ software for calculations. Here each pixel was either 1 or 0 and Z value was obtained by (6).

(6) $$Z=\frac{Observed-Expected}{Standard\;Deviation}$$

Here, the expected and standard deviations were calculated for a random distribution of pixels with the same surface coverage. The number for 1-1 joins are given by (7) with *z_i_* and *z_j_* as box I and j vales (0, 1).

(7) $$0.5\sum_{i}\sum_{j}z_{i}z_{j}$$

## Limitations

Not applicable.

## Ethics Statement

The current work meets the ethical requirements for publication in Data in Brief and does not involve human subjects, animal experiments, or any data collected from social media platforms.

## CRediT authorship contribution statement

**Mona Faraji-Niri:** Conceptualization, Methodology, Data curation, Writing – original draft. **Marc Fransic V. Hidalgo:** Conceptualization, Methodology, Data curation, Writing – original draft. **Geanina Apachitei:** Methodology, Data curation, Writing – review & editing. **Daniela Dogaru:** Data curation. **Michael Lain:** Methodology, Data curation. **Mark Copley:** Resources, Supervision. **James Marco:** Funding acquisition, Resources, Supervision.

## Data Availability

Data of Physical and Electrochemical Characteristics of Calendered NMC622 Electrodes and Lithium-ion Cells at Pilot-Plant Scale Battery Manufacturing (Original data) (Mendeley Data)
